# The dysregulated innate immune response in severe COVID-19 pneumonia that could drive poorer outcome

**DOI:** 10.1186/s12967-020-02646-9

**Published:** 2020-12-03

**Authors:** Mathieu Blot, Jean-Baptiste Bour, Jean Pierre Quenot, Abderrahmane Bourredjem, Maxime Nguyen, Julien Guy, Serge Monier, Marjolaine Georges, Audrey Large, Auguste Dargent, Alexandre Guilhem, Suzanne Mouries-Martin, Jeremy Barben, Belaid Bouhemad, Pierre-Emmanuel Charles, Pascal Chavanet, Christine Binquet, Lionel Piroth, for the LYMPHONIE study group

**Affiliations:** 1 Infectious Diseases Department, Dijon Bourgogne University Hospital , 14 rue Paul Gaffarel, 21079 Dijon, France; 2 Lipness team, INSERM Research Center LNC-UMR1231 and LabEx LipSTIC, University of Burgundy, Dijon, France; 3 Laboratory of virology, Dijon Bourgogne University Hospital, Dijon, France; 4 Department of Intensive Care, Dijon Bourgogne University Hospital, Dijon, France; 5 Clinical Epidemiology unit, INSERM, Dijon, CIC1432 France; 6 Clinical Investigation Center, Clinical Epidemiology/Clinical trials unit, Dijon Bourgogne University Hospital, Dijon, France; 7 Anesthesiology and Critical Care Department, Dijon Bourgogne University Hospital, Dijon, France; 8 Hematobiology, Dijon Bourgogne University Hospital, Dijon, France; 9 Cytometry core facility, University of Burgundy Franche-Comté, Dijon, France; 10 Department of Pneumology, Dijon Bourgogne University Hospital, Dijon, France; 11 Department of Internal Medicine and Clinical Immunology, Dijon Bourgogne University Hospital, Dijon, France; 12 Department of Internal Medicine and Systemic Diseases, Dijon Bourgogne University Hospital, Dijon, France; 13 Geriatrics Internal Medicine Department, Dijon Bourgogne University Hospital, Dijon, France

**Keywords:** COVID-19, Acute respiratory distress syndrome, Pneumonia, Immune response, CXCL10, GM-CSF, Mechanical ventilation

## Abstract

**Background:**

Although immune modulation is a promising therapeutic avenue in coronavirus disease 2019 (COVID-19), the most relevant targets remain to be found. COVID-19 has peculiar characteristics and outcomes, suggesting a unique immunopathogenesis.

**Methods:**

Thirty-six immunocompetent non-COVID-19 and 27 COVID-19 patients with severe pneumonia were prospectively enrolled in a single center, most requiring intensive care. Clinical and biological characteristics (including T cell phenotype and function and plasma concentrations of 30 cytokines) and outcomes were compared.

**Results:**

At similar baseline respiratory severity, COVID-19 patients required mechanical ventilation for significantly longer than non-COVID-19 patients (15 [7–22] vs. 4 (0–15) days; p = 0.0049). COVID-19 patients had lower levels of most classical inflammatory cytokines (G-CSF, CCL20, IL-1β, IL-2, IL-6, IL-8, IL-15, TNF-α, TGF-β), but higher plasma concentrations of CXCL10, GM-CSF and CCL5, compared to non-COVID-19 patients. COVID-19 patients displayed similar T-cell exhaustion to non-COVID-19 patients, but with a more unbalanced inflammatory/anti-inflammatory cytokine response (IL-6/IL-10 and TNF-α/IL-10 ratios). Principal component analysis identified two main patterns, with a clear distinction between non-COVID-19 and COVID-19 patients. Multivariate regression analysis confirmed that GM-CSF, CXCL10 and IL-10 levels were independently associated with the duration of mechanical ventilation.

**Conclusion:**

We identified a unique cytokine response, with higher plasma GM-CSF and CXCL10 in COVID-19 patients that were independently associated with the longer duration of mechanical ventilation. These cytokines could represent the dysregulated immune response in severe COVID-19, as well as promising therapeutic targets.

ClinicalTrials.gov: NCT03505281.

## Background

Severe Acute Respiratory Syndrome Coronavirus 2 (SARS-CoV-2) is responsible for pneumonia with peculiar characteristics [1]. A race against time has been launched to find effective therapies likely to improve outcome in coronavirus disease 2019 (COVID-19).

SARS-CoV-2 infects type I and type II alveolar epithelial cells as well as alveolar macrophages, through binding to angiotensin-converting enzyme 2 (ACE2), triggering a type I interferon (IFN) response, and the release of a myriad of pro-inflammatory cytokines (i.e. interferon (IFN)-γ, interleukin (IL)-1RA, IL-6, IL-8, IL-10, IL-19, monocyte chemoattractant protein (MCP)-1, MCP-2, MCP-3, C-X-C motif chemokine ligand (CXCL)9, CXCL10, CXCL5, tumor-necrosis factor (TNF)-α). Due to the massive T cell stimulation, lower levels of T lymphocytes are observed and all of these abnormalities being associated with disease severity [2–6]. Complement activation, and especially the C5a/C5aR1 axis was also implicated in COVID-19 lung pathology [7]. Accordingly, the description of the so-called cytokine storm has been advocated as the cause of organ dysfunction and death during COVID-19. For now, dexamethasone is the only treatment that has proven to be effective in reducing 28-day mortality in severe COVID-19 patients receiving mechanical ventilation in a randomized clinical trial [8]. These data support the existence of a unique dysregulated immune response that could be one of the most promising therapeutic targets to date.

However, several caveats must be underlined. First, immune pathogenesis is poorly understood, and comparisons of the immune response between COVID-19 patients and patients with pneumonia of other origins are scarce [4, 9]. Second, the relevance of the cytokine storm paradigm is being questioned [10, 11]. We recently reported that COVID-19 patients had lower concentrations of interleukin (IL)-6 compared to non-COVID-19 patients with severe pneumonia [12]. Others showed that mean IL-6 concentrations were nearly 100 times higher in patients with cytokine release syndrome, 27 times higher in patients with sepsis and 12 times higher in patients with ARDS unrelated to COVID-19 [13]. Third, Remy et al. showed that COVID-19 patients display a severe immunosuppressive phenotype [14]. Finally, a delayed type I IFN response is associated with an impeded viral clearance and could promote the accumulation of pathogenic inflammatory monocyte-macrophages resulting in cytokine/chemokine release within the lung [15, 16]. These results are of utmost importance, since we also recently reported that the alveolar viral load is tightly correlated with subsequent severity in COVID-19 acute respiratory distress syndrome (ARDS) [17]. Modulating immunity remains a challenge, and there is a compelling need to identify the dysregulated immune response driving the outcomes observed in COVID-19 patients, in order to find the most relevant therapeutic targets.

Thus, our study aimed to compare clinical and biological characteristics, immune response and outcomes between non-COVID-19 and COVID-19 patients with severe pneumonia.

## Methods

### Study design and participants

The present work is a prospective, exploratory substudy of the ongoing LYMPHONIE trial (ClinicalTrials.gov NCT03505281), initiated in November 2018 at the University Hospital of Dijon-Bourgogne (France). Patients were eligible if they had severe community-acquired pneumonia (CAP): 1) pneumonia (≥ 2 acute signs including cough, purulent sputum, dyspnea, chest pain, temperature < 35 °C or ≥ 38.5 °C, and new radiological pulmonary infiltrate); 2) at least two criteria of the quick-Sequential Organ Failure Assessment (SOFA) score (systolic blood pressure ≤ 100 mm Hg, respiratory rate ≥ 22, Glasgow score < 15) and/or the need for mechanical ventilation (MV) and/or vasopressors; and 3) diagnosed within 48 h following admission. Non-inclusion criteria were: < 18 years, pregnant women, persons under legal protection, decision to limit care, known immune deficiency, chronic disorder known to cause deep lymphopenia (i.e. cirrhosis, lympho- or myeloproliferative syndrome, solid cancer or active systemic lupus), hospitalization for sepsis within the previous 3 months. Non-COVID-19 CAP patients were included until February 20, 2020. COVID-19 patients were eligible if they were tested positive for SARS-CoV-2 by reverse transcriptase-polymerase chain reaction (RT-PCR) on one respiratory sample. Oral consent was obtained from the patient or their legal representative. Approval was obtained from the ethics committee (Comité de Protection des Personnes SUD MEDITERRANEE V; 2017-A03404-49).

### Variables of interest, clinical outcomes, and data collection

Clinical and biological parameters, severity scores (SOFA [18], Simplified Acute Physiology Score (SAPS II) [19] and Pneumonia Severity Index (PSI) [20]) were calculated at the time of inclusion. ARDS was defined according to the Berlin definition [21], and septic shock was defined as persistent hypotension requiring vasopressors and a serum lactate level > 2 mmol/L despite adequate volume resuscitation. Clinical outcomes were recorded up to 30 days after admission, namely: 30-day mortality, hospital- and ICU- length of stay, duration of MV and the occurrence of ventilator-acquired pneumonia (VAP). Dedicated clinical research assistants collected all data using a standardized electronic case report form. Automatic checks were generated for missing or incoherent data.

### Sample collection

Ethylenediamine tetraacetic acid blood (plasma biomarker) and heparin anticoagulated blood (cell stimulation) were obtained after inclusion of the patient (within 48 h of hospital admission, with a diagnosis of severe community acquired pneumonia and according to the inclusion and non-inclusion criteria). Within 4 h following sampling, plasma was collected after centrifugation at 2000 x g for 10 min at 4 °C and stored at −80 °C until use, without freeze–thaw cycle. All samples were collected and stored in the biological resource center of Dijon University Hospital (CRB Ferdinand Cabanne; http://www.crbferdinandcabanne.fr/; NF S96-900 certification).

### Lymphocyte phenotyping

Absolute counts for CD3 + , CD3 + CD4 + ,CD3 + CD8 + , CD3-CD19 + , CD3-CD56 + and/or CD16 + lymphocyte subsets were performed using an AQUIOS CL flow cytometer (Beckman Coulter, Hialeah, FL). The AQUIOS CL is a single platform, fully automated volumetric flow cytometry technology and uses a 488 nm solid state diode laser to measure light diffraction, fluorescence and electronic volume which estimates the relative size of the cells. Whole blood was incubated with the monoclonal antibody reagent followed by no-wash erythrocyte lysis. A ready-to-use mix of antibodies was used. AQUIOS Tetra-1 Panel CD45-FITC/CD4-RD1/CD8-ECD/CD3-PC5 reagent provides identification and enumeration of CD45 + , CD45 + Low SS, and CD3 +/CD4 + , CD3 +/CD8 + , and CD3 + lymphocyte percentages and absolute counts in peripheral whole blood. AQUIOS Tetra-2 + Panel CD45-FITC/(CD56 + CD16)-RD1/CD19-ECD/CD3-PC5 provides identification and enumeration of CD45 + , CD45 + Low SS, and CD3 + , CD3-/CD19 + and CD3-/CD56 + CD16 + lymphocyte percentages and absolute counts in peripheral whole blood. Additionally, both panels provide for CD45 + absolute count and CD45 + Low SS absolute count and percentage. The AQUIOS System Software includes the algorithms and test definitions that provide automated analysis and results for AQUIOS reagents. Normal range (5%–95% reference ranges) values of absolute counts for immune cells and lymphocyte subsets are indicated as Ref. [22, 23].

### Measurement of cytokines

Thirty analytes were quantified in plasma using the Human XL Cytokine Magnetic Luminex^®^ assay (R&D Systems, USA) according to the manufacturer’s instructions: C–C motif chemokine ligand (CCL)2, CCL3, CCL4, CCL5, CCL11, CCL19, CCL20, soluble CD40 ligand, fractalkine, CXCL1, CXCL2, CXCL10, FMS-like tyrosine kinase 3 ligand (FLT3L), granulocyte colony-stimulating factor (G-CSF), granulocyte–macrophage colony-stimulating factor (GM-CSF), granzyme B, interferon (IFN)-α, IL-1α, IL-1β, IL-1RA, IL-2, IL-6, IL-7, IL-8, IL-10, IL-15, IL-33, programmed death-ligand 1 (PDL1), transforming growth factor (TGF)-α, TNF-α, and TNF-related apoptosis inducing ligand (TRAIL). All samples were measured in the same experiment. Briefly, on the day of the assay, plasma was centrifuged again at 16,000x*g* for 4 min immediately prior to use. A twofold dilution with calibrator was used for all samples. The acquisition was performed using Bio-Plex 200 system and analyzed using Bio-Plex Manager^TM^ software (Bio-rad, Hercules, CA). Cytokine concentrations were automatically determined from standard curves and expressed as pg/ml. Samples with values above the ranges were tested again with a 40-fold dilution. All raw data were collected by a data-manager for further analyses in the SAS Software.

### Whole blood leukocyte ex vivo stimulation (WBS)

The standardized functional immunoassay QuantiFERON Monitor^®^ (QFM, Qiagen) was used according to the manufacturer’s instructions. Within 3 h after blood sampling, one milliliter of whole blood was incubated at 37 °C for 20 ± 1 h with a QFM LyoSphere containing anti-CD3 T-cell receptor ligand and R848 (TLR7/8 ligand), or without LyoSphere (non-stimulated blood). Plasma was harvested after centrifugation at 4000 rpm for 10 min and stored at −80 °C until use. Whole blood leukocyte production of IFN-γ (IU/ml) upon stimulation was measured using ELISA (Qiagen), and fifteen other analytes using the Human Th9/Th17/Th22 Discovery Luminex^®^ assay (R&D Systems, USA): CD40 ligand, GM-CSF, IL-1β, IL-2, IL-4, IL-5, IL-6, IL-10, IL-12, IL-13, IL-15, IL-17A, IL-33, TNF-α, CCL20. All samples were measured the same day by the same person and using the same kit. Samples with values above the ranges were tested again with further dilution. The cytokine production after stimulation was expressed as the difference of concentrations between plasma from stimulated blood and those from non-stimulated blood.

As a reference, we used samples from 7 control patients included in the Pneumochondrie study (ClinicalTrials.gov NCT03955887) and who underwent QuantiFERON Monitor^®^ assay in the same conditions. The control population consisted of outpatients without fever during the previous 15 days and who underwent bronchoalveolar lavage for a non-infectious condition [24]. Samples were conditioned and measured in the same way as for the LYMPHONIE study.

### Statistical analysis

Data were described according to COVID-19 status (i.e. non-COVID-19 vs COVID-19). Continuous variables were expressed as mean ± standard deviation (SD) or median and inter-quartile range (IQR), according to their distribution, and categorical variables as frequencies and percentage. Univariate comparisons were performed using Student’s test for means, Wilcoxon Mann–Whitney test for medians and IQRs and Chi square test (or Fisher’s exact test when appropriate) for percentages. Cytokines with p < 0.05 were presented by boxplots to visualize potential associations with COVID-19 status.

Principal component analysis (PCA) was used to identify potentially significant patterns of 64 variables: clinical characteristics and outcomes (n = 6), biological findings (n = 13), plasma cytokines (n = 30), cytokine production upon ex vivo stimulation (n = 15). PCA identifies factors, called principal components, that induce the most variation in the overall data [25]. These factors can be expressed as a linear combination of the correlated original variables (OVs). By inversing these formulas, we can express each OV as a linear combination of the factors and coefficients defining these linear combinations are interpreted as correlation coefficients. Moreover, each factor describes a percent of variation in the OVs. The number of factor to retain was determined using the scree plot [26] and the clinical interpretability of factors [27]. Finally, patients OVs data can be projected on the plans defined by the retained factors, which allows observing patient’s patterns in a two-dimensional plot.

Spearman correlations were computed between cytokines and the most pertinent clinical outcomes associated with Covid-19 status in univariate analyses and PCA patterns. To account for potential confounders, we constructed multivariable linear regressions, with the MV duration as an outcome, for each selected cytokine. adjusted for age, COVID-19 status and either SOFA score (model 1) or PaO_2_:FiO_2_ (model 2). The interaction between COVID-19 status and cytokines was systematically tested. Absence of serial correlation and heteroscedasticity were assessed using the DW statistic [28] and the White test [29] respectively. The proportion of variance explained by the models was quantified using the R^2^ coefficient. Measures of association are expressed as mean differences ± standard error (SE). A p-value < 0.05 was considered statistically significant. Analyses were performed using SAS version 9.4 (SAS Institute Inc., Cary, NC, USA).

## Results

### Characteristics of the study population

Sixty-three patients were enrolled (36 in the non-COVID-19 group, and 27 in the COVID-19 group). Bacterial, viral or mixed etiologies were proven in 10 (28%), 10 (28%) and 3 (8%) patients from the non-COVID-19 group, respectively (Additional file 1: Table S1). Mean age was marginally lower in the COVID-19 group as compared to the non-COVID-19 group (62.5 ± 10.9 vs. 68.0 ± 13.0; p = 0.07). Other demographic and comorbidity data were not statistically different between the two groups (Table 1). Although PaO_2_:FiO_2_ ratio and SOFA score were not different between groups (p = 0.35 and p = 0.52, respectively), fewer COVID-19 patients had septic shock (0 vs. 11 (31%); p = 0.0015), and arterial lactates (p = 0.01), serum creatinine (p = 0.02), and NT-proBNP (p = 0.0003) were all lower in COVID-19 patients (Table 1). White blood cells counts did not differ significantly between groups (Table 1).

**Table 1:** Baseline characteristics and outcomes of the study population (LYMPHONIE study, 2018–2020)

	Normal range	Study group		P
		Non-COVID-19 N = 36	COVID-19 N = 27	
Demographics				
Age (years), mean ± SD		68.0 ± 13.0	62.5 ± 10.9	0.07
Male sex, n (%)		29 (81%)	17 (63%)	0.12
Body-mass index (kg/m^2^), mean ± SD		29.1 ± 7.1	30.7 ± 8.1	0.43
Chronic comorbidities				
Cardiovascular disease, n (%)		12 (33%)	5 (19%)	0.25
Pulmonary disease, n (%)		12 (33%)	5 (19%)28	0.25
Chronic renal disease, n (%)		2 (6%)	1 (4%)	0.73
Cerebrovascular disease, n (%)		5 (14%)	3 (11%)	0.74
Diabetes mellitus, n (%)		10 (28%)	2 (7%)	0.28
Tobacco use, n (%)		10 (28%)	2 (7%)	0.055
Charlson score, mean ± SD		1.5 ± 2.0	0.9 ± 0.9	0.12
Severity at hospital admission				
Septic shock, n (%)		11 (31%)	0	0.0015
ARDS, n (%)		23 (64%)	25 (93%)	0.015
Pneumonia Severity Index, mean ± SD		117.8 ± 38.6	94.2 ± 27.1	0.006
SAPS II, mean ± SD		23.8 ± 9.9	19.4 ± 9.4	0.08
SOFA score, mean ± SD		7.2 ± 3.6	6.7 ± 2.0	0.52
Biological findings at admission				
ASAT (IU/l), mean ± SD	15–37	86.3 ± 92.4	86.2 ± 54.6	0.99
Serum Creatinine (μmol/l), mean ± SD	59–104	132.9 ± 93.3	90.2 ± 40.7	0.02
NT-ProBNP (pg/ml), mean ± SD	<125	5687 ± 7694	2225 ± 6257	0.05
PaO_2_:FiO_2_ (mm Hg), mean ± SD	≥400	123.7 ± 54.9	136.2 ± 49.8	0.35
Arterial pH (mm Hg), mean ± SD	7.35–7.45	7.35 ± 0.11	7.40 ± 0.07	0.07
Serum Bicarbonate (mmol/l), mean ± SD	20–29	24.0 ± 5.1	24.6 ± 3.1	0.59
Lactate level (mmol/l), mean ± SD	0.5–2.0	2.6 ± 1.9	1.7 ± 0.7	0.01
C-reactive protein (mg/l), mean ± SD	<3.2	259.9 ± 156.8	172.8 ± 62.9	0.004
Procalcitonin (μg/L), mean ± SD	<0.10	32.4 ± 61.9	2.6 ± 6.6	0.007
Immune cells				
Leukocytes (x10^6^/l), mean ± SD	3.8–9.5	12.2 ± 6.4	10.8 ± 5.7	0.38
Neutrophils (x10^6^/l), mean ± SD	1.7–5.8	11.3 ± 5.5	9.4 ± 5.6	0.18
Lymphocytes (x10^6^/l), mean ± SD	1.07–4.03	0.64 ± 0.40	0.78 ± 0.38	0.16
Monocytes (x10^6^/l), mean ± SD	0.2–0.7	0.61 ± 0.46	0.44 ± 0.25	0.05
Lymphocyte subsets				
CD3 + (/μl), mean ± SD	605–2460	360.8 ± 281.0	443.6 ± 256.1	0.16
CD4 + (/μl), mean ± SD	493–1666	241.4 ± 187.5	288.1 ± 264.0	0.44
CD8 + (/μl), mean ± SD	224–1112	111.6 ± 100.9	145.1 ± 137.1	0.38
CD4/CD8 ratio, mean ± SD	0.5–6.4	2.7 ± 1.7	2.7 ± 2.1	0.97
NK cells (/μl), mean ± SD	73–654	103.8 ± 83.7	103.1 ± 87.7	0.12
NKT cells (/μl), mean ± SD	NA	27.3 ± 33.3	44.6 ± 67.4	0.19
B cells (/μl), mean ± SD	72–520	95.6 ± 81.8	123.7 ± 78;4	0.17
Treatments				
Antibiotic multitherapy, n (%)		29 (81%)	17 (63%)	0.12
Corticosteroids, n (%)		16 (44%)	16 (59%)	0.24
Hydroxychloroquine, n (%)		0	10 (37%)	
Remdesivir, n (%)		0	3 (11%)	
Invasive mechanical ventilation, n (%)		24 (67%)	23 (85%)	0.09
ECMO, n (%)		0	1 (4%)	0.43
Renal replacement therapy, n (%)		5 (14%)	0	0.065
Vasopressors, n (%)		19 (53%)	19 (70%)	0.16
Outcomes at 30 days				
ICU admission, n (%)		32 (89%)	274 (100%)	0.12
Median ICU length of stay (days) (IQR)		13 (4–20)	20 (12–29)	0.0274
Median days of mechanical ventilation (IQR)		4 (0–15)	15 (7–22)	0.0049
Median hospital length of stay (days) (IQR)		21 (13–30)	29 (20–30)	0.087
Ventilatory acquired pneumonia				0.001
0 event, n (%)		29 (81%)	11 (41%)	
1 event, n (%)		2 (6%)	11 (41%)	
2 events, n (%)		5 (14%)	5 (19%)	
Median days of antibiotic treatment(IQR)		12 (8–21)	15 (8–23)	0.48
30 day mortality, n (%)		2 (6%)	1 (4%)	1

*ARDS* acute respiratory distress syndrome, *SAPS II* Simplified Acute Physiology Score II, *SOFA* Sequential Organ Failure Assessment, *ASAT* aspartate aminotransferase, *NT-proBNP* N-Terminal *Fragment* of the prohormone *Brain*-Type Natriuretic Peptide, *PaO*_*2*_*:FiO*_*2*_ arterial pressure of oxygen/oxygen inspiratory fraction, *NK* Natural killer, *ECMO* extracorporeal membrane oxygenation, *ICU* intensive care unit

### COVID-19 patients had a significantly longer duration of mechanical ventilation

At baseline respiratory severity, COVID-19 patients had a significantly longer duration of MV (15 [7–22] vs. 4 [0–14.5]; p = 0.0049), and ICU stay (p = 0.0274) and higher rate of ventilator-acquired pneumonia (p = 0.001) than non-COVID-19 patients (Table 1). The 30-day mortality rate was 6% (n = 2) in the non-COVID-19 group and 4% (n = 1) in the COVID-19 group (p = 1.00).

### COVID-19 patients displayed a unique plasma cytokine response pattern

**Fig. 1:**
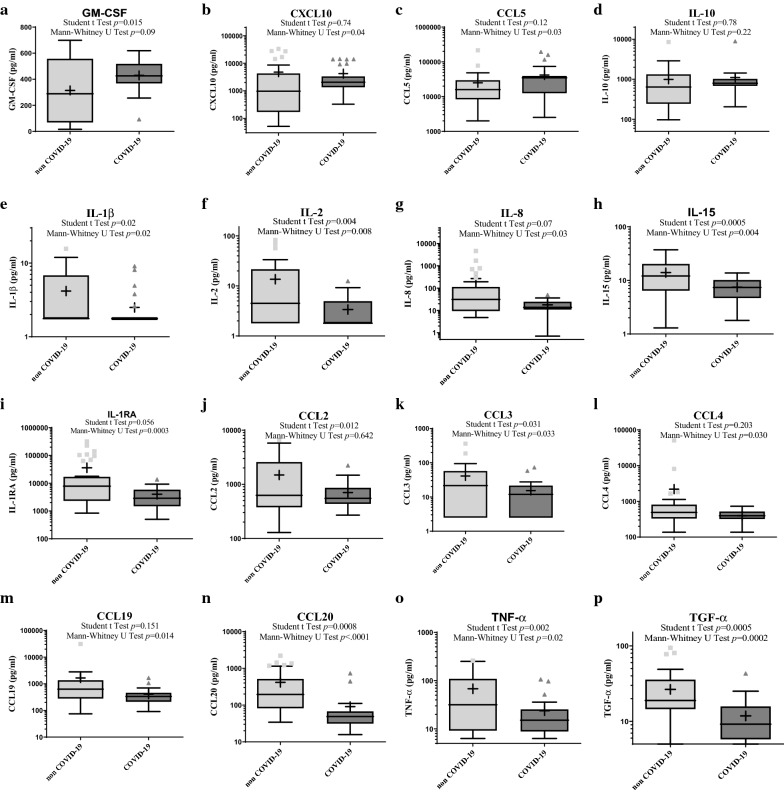
Box plot showing plasma concentrations of cytokines in non-COVID-19 and COVID-19 patients. Plasma concentration of cytokines was measured within 48 h of hospitalization in 36 non-COVID-19 and 27 COVID-19 patients with severe pneumonia. For each cytokine, *p*-values from both Student t and Wilcoxon-Mann–Whitney U tests are indicated and the difference was considered significant if at least one was < 0.05. *CCL* C–C motif chemokine ligand, *COVID-19* coronavirus disease 2019, *IL* interleukin, *GM-CSF* Granulocyte–macrophage colony-stimulating factor, *TNF* tumor necrosis factor, *TGF* transforming growth factor (LYMPHONIE study, 2018–2020)

COVID-19 patients had higher plasma CXCL-10 and CCL5 and marginally higher GM-CSF (Fig. 1). However, we observed lower plasma levels of FLT3L, G-CSF, CXCL1, IL-1β, IL1-RA, IL-2, IL-6, IL-8, IL-15, CCL2, CCL4, CCL19, CCL20, TGF-α and TNF-α, and a non-significant difference for plasma levels of sCD40-Ligand, CX3CL1, Granzyme B, CXCL2, INF-α, IL-1α, IL-7, IL-10, IL-33, PD-L1 and TRAIL (Fig. 1, Additional file 1: Table S2).

### COVID-19 patients displayed similar T-cell exhaustion to non-COVID-19 patients

**Fig. 2:**
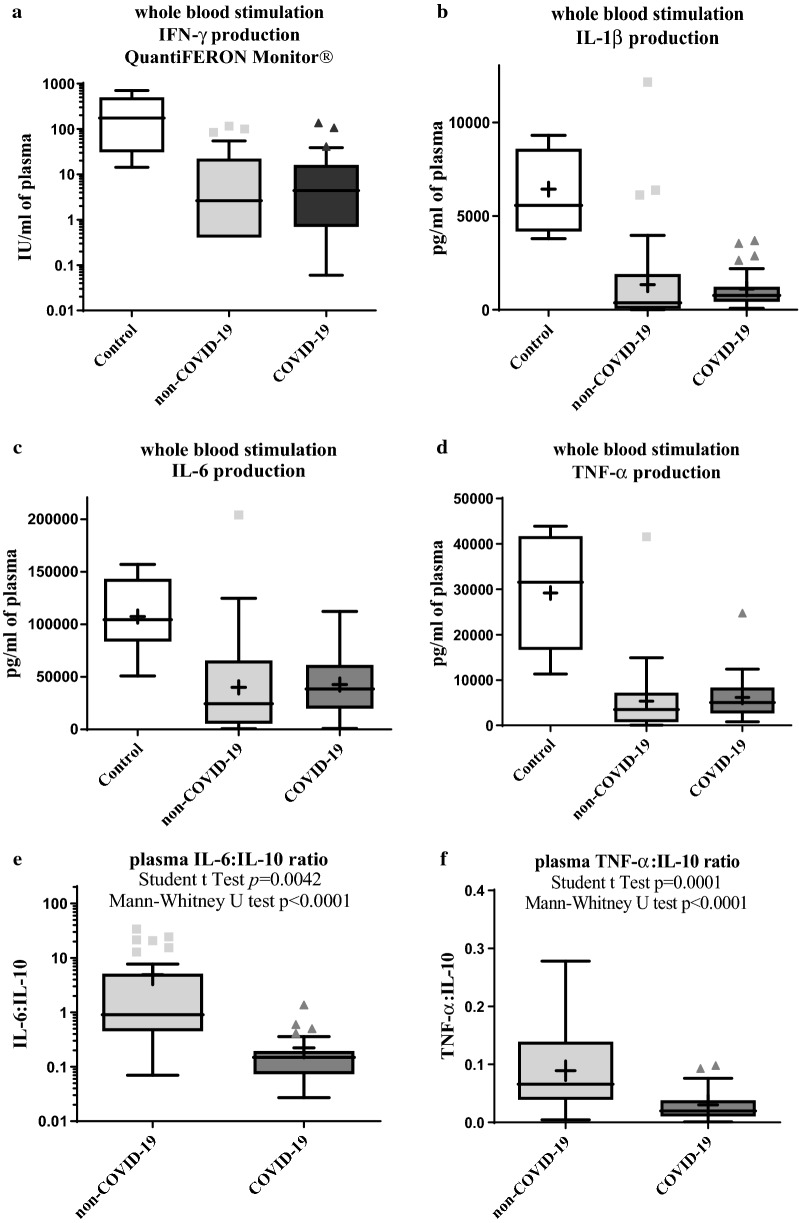
Immune-suppression phenotype and inflammatory/anti-inflammatory balance in COVID-19 and non-COVID-19 patients. Boxplot showing cytokine production (IFN-γ (**a**), IL-1β (**b**), IL-6 (**c**), TNF-α (**d**)) of blood leukocytes on ex vivo stimulation (CD3/TLR7-8 agonists), using a standardized test (QuantiFERON Monitor^®^) within 48 h of hospitalization in non-COVID-19 (n = 36) and COVID-19 (n = 27) patients. As a reference, the test was performed in 7 non-infected control patients included in the Pneumochondrie study (NCT03955887) [24]. Boxplot depicting IL-6:IL10 (d) and TNF-α:IL-10 ratios in non-COVID-19 and COVID-19 patients. *COVID-19* coronavirus disease 2019, *IL* interleukin, *TNF* tumor necrosis factor, *WBS* whole blood stimulation (LYMPHONIE study, 2018–2020)

On ex vivo stimulation with CD3 and TLR7/8, T-cells from patients with severe CAP (non-COVID-19 and COVID-19), as compared to non-infectious controls, displayed a significantly decreased production of INF-γ, TNF-α, IL-1β, IL-2, IL-4, IL-5, IL-6, IL-10, IL-12, IL-13, IL-15, IL-17A and IL-33, with no difference between COVID-19 and non-COVID-19 groups (Fig. 2a–d, Additional file 1: Table S3). In addition, the IL-6:IL-10 and TNF-α:IL-10 ratios were significantly lower in COVID-19 patients as compared to non-COVID-19 patients (p = 0.0042 and p = 0.0001, respectively), driven by lower IL-6 and TNF-α levels in COVID-19 patients with similar levels of IL-10 between groups (Fig. 2e–f).

### PCA identifies two patterns linking immune response and outcomes, with a clear distinction between non-COVID-19 and COVID-19 patients

**Fig. 3:**
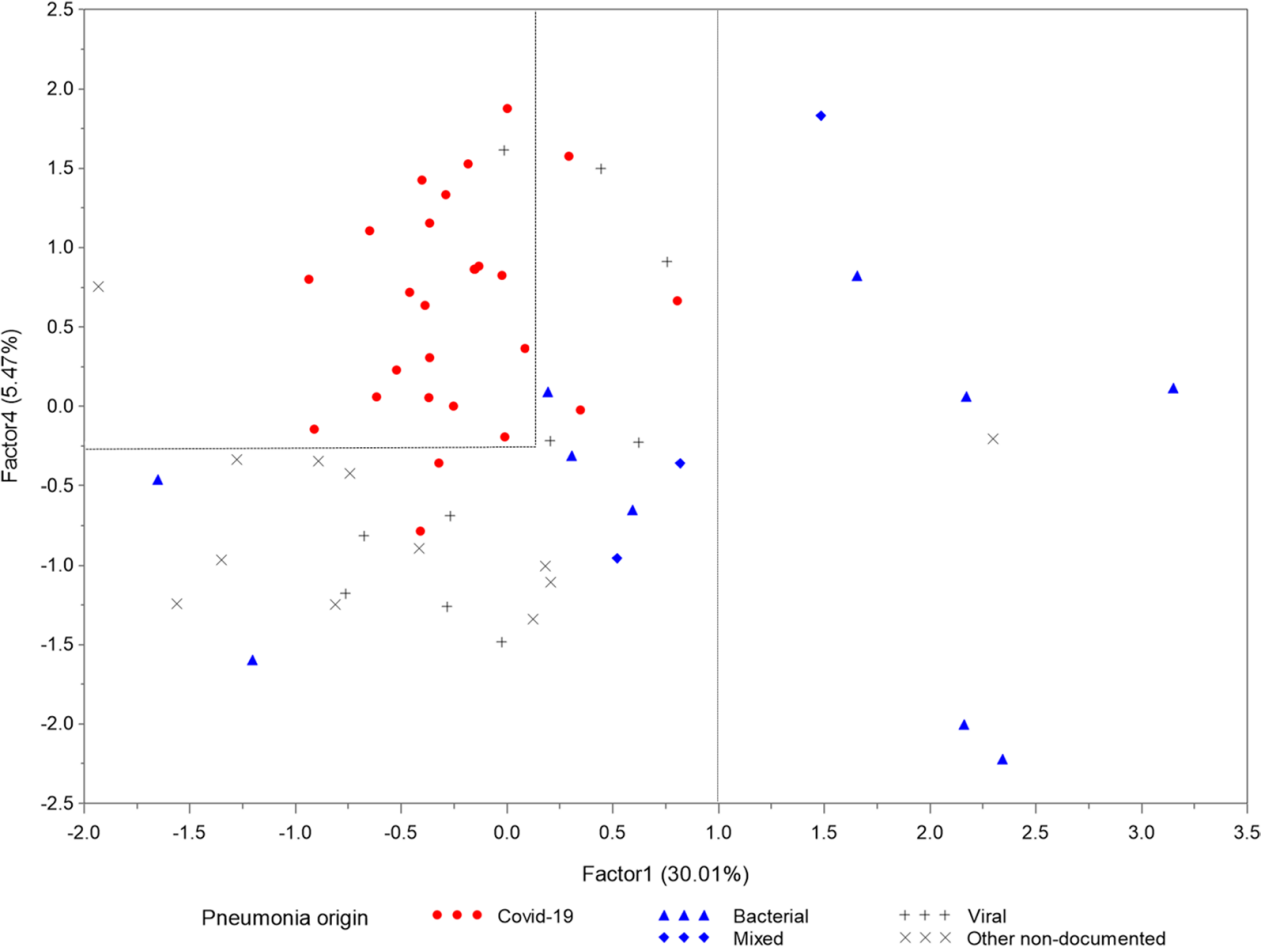
Two-dimensional score plot of principal component analysis according to pneumonia etiology. Principal component analysis (PCA) was used to identify potentially significant patterns of 65 variables (clinical (n = 8), biological (n = 12), plasma cytokines (n = 30), cytokine production on ex vivo stimulation (n = 15)) from 63 patients with severe pneumonia (non-COVID-19 (n = 36), COVID-19 (n = 27)). Factors 1 and 4 were used to build a two-dimensional score plot of PCA and COVID-19 patients (red circles) and non-COVID-19 patients (bacterial (blue triangles), mixed (blue diamonds), viral (grey crosses) and other non-documented severe community acquired pneumonia (grey Xs)) were represented. (LYMPHONIE study, 2018-2020)

In PCA, four factors were retained for interpretation, which together accounted for 53.81% of all the information obtained by using the whole 64 available variables (clinical characteristics and outcomes (n = 6), biological findings (n = 13), plasma cytokines (n = 30), cytokine production upon ex vivo stimulation (n = 15)). Two of the four factors in the final pattern were associated with outcomes (Table 2, Additional file 1: Fig. S1). Factor 1 associated [1] baseline severity and extra-respiratory organ dysfunction (SOFA and PSI scores, lactate, creatinine and NT-ProBNP levels which were higher in non-COVID-19, except for SOFA score), [2] “classical” inflammatory mediators (i.e. CXCL1, CXC2, IL-1β, IL-2, IL-6, IL-8, TNF-α) and (3) “T-cell exhaustion (inverse correlation with lymphocyte count, and production of 15 cytokines upon ex vivo stimulation of whole blood with anti-CD3 and TLR7/8 ligands). Factor 4 associated: [1] outcomes (duration of MV, ICU and hospital length of stay, which were higher in COVID-19 patients at 30 days) and (2) only GM-CSF, CXCL10 and INF-α levels (Table 2). The projection of patient data onto the directions defined by Factors 1 and 4 showed a clear separation between COVID-19 and non-COVID-19 patients. By plotting patients according to different origins of pneumonia, we observed that most of the non-COVID-19 patients with proven bacterial (or mixed) origin had high Factor 1 values, while COVID-19 patients had high Factor 4 and moderate Factor 1 values (Fig. 3). Planes defined by factors 2 and 3 did not enable any such discrimination between patients according to COVID-19 status.

**Table 2:** Principal component analysis (PCA) factor pattern correlation (LYMPHONIE study, 2018-2020)

Factor Pattern	Factor1	Factor2	Factor3	Factor4
Clinical features and outcomes				
Body mass index	–	0.319	–	–
Pneumonia Severity Index	0.393	–	–	–
SOFA score	0.571	–	0.382	–
ICU length of stay	–	–	0.333	0.686
Mechanical ventilation duration	–	–	0.331	0.70
Hospital length of stay	–	−0.303	0.344	0.585
Biological findings and immune cells				
PaO_2_:FiO_2_	–	–	–	–
Lactate level	0.675	–	–	–
C-reactive protein	0.506	–	–	−0.302
Procalcitonin	0.579	–	0.375	−0.442
Serum Creatinine	0.623	–	0.318	−0.344
NT-ProBNP	0.596	–	–	–
Leukocytes	–	–	−0.644	–
Neutrophils	–	–	−0.622	–
Monocytes	−0.472	–	−0.322	−0.317
Lymphocytes	−0.567	–	–	–
CD3 + T Lymphocytes	−0.587	–	–	–
CD4 +/CD8 + T Lymphocytes	–	–	–	–
NK cells	–	–	–	–
Cytokine plasma concentrations				
sCD40-Ligand	0.525	0.472	−0.396	–
FLT3L	0.699	0.466	–	–
CX3CL1	0.732	–	–	–
G-CSF	0.553	0.316	–	–
GM-CSF	0.615	–	–	0.501
Granzyme B	0.618	0.478	–	–
CXCL1	0.534	–	–	–
CXCL2	0.432	–	–	–
INF-α	0.300	0.328	–	0.308
IL1-α	0.347	0.453	−0.561	–
IL1-ß	0.672	0.492	–	–
IL1-RA	0.628	–	0.343	–
IL-2	0.648	0.344	–	–
IL-6	0.588	0.552	–	–
IL-7	0.419	–	−0.548	–
IL-8	0.491	–	0.301	–
IL-10	0.477	0.312	–	–
IL-15	0.815	–	–	–
IL-33	0.307	0.402	–	–
CXCL10	0.635	–	–	0.35
CCL2	0.696	–	0.453	–
CCL3	0.589		0.406	–
CCL4	0.361	–	0.409	–
CCL20	0.737	–	–	–
CCL19	0.498	0.544	–	–
PD-L1	0.787	–	–	–
CCL5	–	0.318	−0.582	–
TGF-α	0.401	0.465	−0.467	–
TNF-α	0.837	–	–	–
TRAIL	−	–	−0.36	–
Cytokine production of blood leukocytes on ex vivo stimulation				
sCD40-Ligand (WBS)	−0.809	0.481	–	–
GM-CSF (WBS)	−0.65	0.45	–	–
INF-γ (WBS)	−0.426	0.309	–	–
IL1-ß (WBS)	−0.624	0.487	–	–
IL-2 (WBS)	−0.609	0.414	–	–
IL-4 (WBS)	−0.432	–	0.314	–
IL-5 (WBS)	−0.414	0.340	–	
IL-6 (WBS)	−0.671	0.565	–	
IL-10 (WBS)	−0.499	0.347	–	
IL-12 (WBS)	−0.772	0.474	–	
IL-13 (WBS)	−0.686	0.414	–	
IL-15 (WBS)	−0.824	0.399	–	
IL-17A (WBS)	−0.472	0.349	–	
IL-33 (WBS)	−0.805	0.485	–	
TNF-α (WBS)	−0.617	0.471	–	

The table shows results of principal component analysis (PCA) including 64 variables (clinical characteristics and outcomes (n = 6), biological findings (n = 13), plasma cytokines (n = 30), cytokine production upon ex vivo stimulation (n = 15)). For clarity, we present only results for magnitude of the loading of at least 0.3

*SOFA* Sequential Organ Failure Assessment, *PaO*_*2*_*:FiO*_*2*_ arterial pressure of oxygen/oxygen inspiratory fraction, *ICU* intensive care unit, *NT-proBNP* N-Terminal *Fragment* of the Prohormone *Brain*-Type Natriuretic Peptide, *NK* Natural killer, *WBS* whole blood stimulation

### Plasma GM-CSF, CXCL10 and IL-10 were independently associated with the duration of mechanical ventilation

As the particular severity of COVID-19 patients was represented by a significantly longer duration of MV, we investigated whether immune response could explain this poor outcome. We observed a significant correlation between the duration of MV and GM-CSF (p < 0.0001), IL-10 (p < 0.0001), CXCL10 (p < 0.0001), CCL-2 (p = 0.001), CX3CL1 (p = 0.0233), and Granzyme B (p = 0.0143) (Additional file 1: Table S4). Based on all these results, we performed a multivariate linear regression to identify factors associated with the duration of MV in the first 30 days, using two models (i.e. SOFA score (model 1) or PaO_2_:FiO_2_ ratio (model 2) as the variable of adjustment to account for severity) (Table 3). Only GM-CSF was independently associated with a longer duration of MV in both models. We estimated an excess of 22.11 ± 8.36 min of MV per increase of 1 pg/mL of GM-CSF (p = 0.0105, model 1; and p < 0.0001, model 2). Interleukin-10 and CXCL10 were independently associated with a longer duration of MV only when adjusted for respiratory severity (model 2; p = 0.0359 and p = 0.049 respectively) (Table 3). No significant interaction was found between these cytokines and COVID-19 status. Autocorrelation and heteroscedasticity were non-significant in all models.

**Table 3:** Multivariate linear regression factors associated with duration of mechanical ventilation (variation in minutes) in the first 30 days of hospitalization among 63 patients with severe pneumonia (LYMPHONIE study, 2018-2020)

Model Variable effect	GM-CSF model 1			GM-CSF model 2			CXCL10 model 1			CXCL10 model 2			IL-10 model 1			IL-10 model 2		
	Mean diff	(± SE)	p	Mean diff	±SE	p	Mean diff	±SE	p	Mean diff	±SE	p	Mean diff	±SE	p	Mean diff	±SE	p
GM-CSF (per 1 pg/ml increase)	*22.1*	*(*± *8.4)*	*0.011*	*32.7*	*(*± *7.5)*	<*.0001*		NE			NE			NE			NE	
CXCL10 (per 100 pg/ml increase)		NE			NE		24.2	(± 23.2)	0.302	*46.4*	*(*± *23.1)*	*0.049*		NE			NE	
IL-10 (per 10 pg/ml increase)		NE			NE			NE			NE		13.7	(± 10.8)	0.208	*23.8*	*(*± *11.1)*	*0.036*
Age (per additional year)	−19.0	(± 121.2)	0.876	42.0	(± 121.1)	0.730	−1.8	(± 127.0)	0.989	69.3	(± 135.1)	0.61	−29.9	(± 129.4)	0.8178	30.19	(± 137.4)	0.827
COVID-19 (Yes vs No)	*8,149.7*	*(*± *3200.6)*	*0.014*	*7,633.2*	*(*± *3142.4)*	*0.018*	*1,113*	*(*± *3,142.4)*	*0.001*	*11.7*	*(*± *3.3)*	*0.0009*	*10.7*	*(*± *3,2)*	*0.001*	*11,12*	*(*± *3,341.8)*	*0.002*
SOFA (for each additional point)	*1,667.8*	*(*± *542.5)*	*0.003*		NE		*2,080.9*	*(*± *551.14)*	*0.0004*		NE		*2,139.4*	*(*± *524)*	*0.0001*		NE	
PaO_2_:FiO_2_ (per 1 point increase)	NE			−*90.2*	*(*± *27.8)*	*0.002*	NE			−*82.6*	*(*± *31.4)*	*0.011*	NE			−*93.0*	*(*± *30.8)*	*0.004*
R^2^	41.46%			42.4%			35.61%			28.33%			36.18%			29.0%		

*GM-CSF* Granulocyte–macrophage colony-stimulating factor, *IL* interleukin, *CXCL* C-X-C motif chemokine ligand, *NE* not entered in the model, *SOFA* Sequential Organ Failure Assessment, *PaO2:FiO2* arterial pressure of oxygen/oxygen inspiratory fraction, *SE* standard error

## Discussion

In this study, we identify a dysregulated cytokine production of GM-CSF and CXCL-10 in COVID-19 patients that was independently associated with the duration of MV which represents the distinctive poorer outcome observed in severe COVID-19 patients.

COVID-19 pneumonia is unique in comparison with pneumonia of other origins, with, in particular, sudden deterioration 7–9 days after onset of symptoms, severity of hypoxemia that contrasts with the relatively well-preserved lung mechanics, and the protracted nature of ARDS [30, 31], as observed in our study. The beneficial effect of dexamethasone in the most severe forms of COVID-19 argues for a dysregulated immune response that mediates lung injury and outcome [8]. To date, the characteristics of the immune response in COVID-19 have not been completely elucidated. The terms “cytokine storm” and “macrophage activation syndrome” have been widely adopted to explain the immunopathogenesis, since the release of myriad inflammatory mediators is correlated with disease severity [4, 32]. In our study, we first showed that despite similar respiratory severity, plasma concentrations of numerous cytokines characterizing the “cytokine storm” (i.e. IL-1β, IL-6, IL-8, IL-15, TNF-α, CCL2, CCL4, CCL19, CCL20, TGF-α) were lower in COVID-19 compared to non-COVID-19 patients. These findings contrast with the results of McElvaney et al., showing that plasma IL-6 levels were higher in COVID-19, compared to non-COVID-19 patients (4), but are in line with Sinha’s retrospective observations (10). Conversely, based on a standardized functional immune-assay, we found that patients with severe pneumonia (whether COVID-19-related or not) displayed severe alterations of T-cell functionality on ex vivo CD3 and TLR7/8 stimulation. However, we observed a more unbalanced inflammatory/anti-inflammatory cytokine response in COVID-19 patients, as reflected by the IL-6:IL-10 and TNF-α:IL-10 ratios. Our results and those of Remy et al. [14] clearly challenge the classical paradigm of “cytokine storm”-mediated inflammation and show a markedly immunosuppressive phenotype in COVID-19 patients rather than hyperinflammation.

However, we identified a dysregulated immune response that was independently associated with the peculiar longer duration of MV observed in severe COVID-19 patients. PCA analysis, including a comprehensive study of inflammatory and anti-inflammatory immune responses and outcomes, identified two interesting patterns that clearly distinguish non-COVID-19 from COVID-19 patients. COVID-19 patients presented a unique phenotype associating higher levels of GM-CSF and CXCL10 and a longer duration of MV. In addition, multivariate regression analysis confirmed that GM-CSF, CXCL10, and also IL-10 were all independently associated with the duration of MV after adjustment for potential confounders. Based on this comprehensive analysis, we hypothesize that these cytokines could represent part of the dysregulated immune response driving the prolonged need for MV in COVID-19 patients.

GM-CSF is secreted by epithelial cells from injured tissue or leukocytes, to induce survival, proliferation and/or differentiation of myeloid cells [33], playing a critical role in regulating microbial defense [34]. However, as a consequence of aberrant Th-1 cell activation and inflammatory monocytes [35], aberrant production of GM-CSF may result in excessive inflammation and tissue damage, mainly by macrophage M1 polarization and overactivation [36]. CXCL10 is a pro-inflammatory Th1-chemokine driving migration to the site of infection of Th-1 T-cells, monocytes and neutrophils that express its receptor CXCR3 [37]. Production of CXCL10 has already been shown to be increased in SARS-CoV-1 [38]. Plasma concentrations of CXCL10 were recently reported to predict disease progression in COVID-19 [39, 40]. Finally, Ichikawa et al. showed that blocking the CXCL10-CXCR3 signaling pathway in viral and non-viral ARDS preclinical models improved survival [41]. High levels of interleukin-10 were also observed in our study, whether COVID-19-related or not, and were independently associated with the duration of MV. However, no statistically significant association between the COVID-19 status and IL-10 was observed, which may be explained either by the fact that IL-10 is not the only or main driver of the length of MV in COVID-19 patients, either by a lack of statistical power, or both. As recently described, concurrent immune suppression and hyperinflammation are a hallmark of the pathogenesis in non-COVID-19 CAP, and argue against two distinct phases of host response [42]. Nevertheless, in COVID-19 patients, we observed higher IL-6:IL-10 and TNF-α:IL-10 ratios that could reflect an unbalanced overproduction of IL-10. IL-10 can be produced by most cells of innate and adaptative immune system, including lymphoid and myeloid cells, resulting in pleiotropic immunosuppressive functions (e.g. inhibition of the release of pro-inflammatory mediators, inhibitory effects on T cells and monocytes/macrophages…) [43]. As for CXCL10, it can be advocated that an initial more robust TH1 response with monocytes/macrophage over activation in COVID-19 patients, as compared to non-COVID-19 patients, could lead to a subsequent IL-10 overproduction in order to limit T cell responses. The role of IL-10, either beneficial or deleterious remains a difficult issue. However, IL-10-mediated immune suppression could drive the onset of secondary infectious complications and morbi-mortality, especially since 59% of COVID-19 patients presented at least 1 VAP event.

Our results are in line with those of Hue et al. that recently identified a plasma chemokine signature in COVID-19 ARDS patients (CXCL10, GMCSF and IL-10) which was associated with mortality (9). In addition, we also recently reported that both plasma and alveolar CXCL10 concentrations were independently associated with the duration of mechanical ventilation in COVID-19 ARDS patients [24].

Corticosteroids were shown to improve survival in severe COVID-19 patients in a recent therapeutic trial [8], and this may be linked to a decrease in CXCL10 levels [40], via the inhibition of the Th-1 pathway. Since corticosteroids have many side effects, targeted therapies likely to dampen the dysregulated immune response in COVID-19 are urgently needed. Specifically, GM-CSF blockade (e.g. with lenzilumab) is increasingly being considered as a promising therapy in COVID-19 [33, 36] and is under investigation in a phase III clinical trial (NCT04351152). In addition, CXCL10 blockade (e.g. Eldelumab/MDX-1100) may also represent an attractive therapy likely to dampen the dysregulated immune response that could be driving the duration of MV.

Dampening inflammation in a context of high immune suppression is not always a hazardous route. During chimeric antigen receptor T (CAR-T) cell therapy, GM-CSF inhibition reduces cytokine release syndrome and neuro-inflammation, but enhances antitumoral CAR-T cell function [44]. In addition, an IL-6 blocker could partially rescue immune dysregulation caused by SARS-CoV-2 (2). These considerations are of utmost importance, since we reported that COVID-19 ARDS patients had a persistent alveolar SARS-CoV-2 viral load that correlates with severity [17]. Combined therapies associating immunomodulatory and antiviral agents are the most promising strategy likely to improve outcome in COVID-19 patients.

This study has several limitations. The statistical analysis suffers from a lack of power given the large number of variables studied and the small sample size. Then, we only used one approach (cytokine production after stimulation with anti-CD3 and TLR7/8 ligand) to measure T-cells exhaustion phenotype. However, it would have been also important to measure several phenotypic markers of exhaustion, namely PD-1, LAG-3, TIM-3 and CTLA-4. However, it was an exploratory study in the context of a pandemic emergency. Additionally, we used several statistical methods to assess the association between COVID-19 status and immune targets. Comparisons of immune response between non-COVID-19 and COVID-19 severe pneumonia are still scarce in the literature, even though they are mandatory to understand the distinctive pathogenesis of severe forms of COVID-19.

## Conclusion

Higher plasma GM-CSF and CXCL10 were reported in COVID-19 patients and could represent the dysregulated immune response in COVID-19 patients driving the longer duration of mechanical ventilation in severe pneumonia. These cytokines could be considered as promising therapeutic targets.

## Supplementary information

**Additional file 1: Table S1.** Microbiological etiologies of severe pneumonia from 63 patients**. Table S2.** Plasma cytokine concentrations**. Table S3.** Blood leukocyte cytokine production on ex vivo stimulation**. Table S4.** Spearman correlation between plasma cytokine concentrations and severity (PaO_2_:FiO_2_ ratio, SOFA score) or outcome (duration of mechanical ventilation)**. Table S5.** Spearman correlation between blood leukocyte cytokine production on ex vivo stimulation (Whole Blood Stimulation (WBS)) and severity (PaO_2_:FiO_2_ ratio, SOFA score) or outcome (duration of mechanical ventilation). **Figure S1.** Principal component analysis screen plot (retained dimension = 4) Cattell scree plot criterion retained the first four independent factors; which were clinically interpretable and together preserved 53.47% of all the information contained in the 65 correlated original variables.

## Data Availability

all data are available on demand.

## Abbreviations

ACE2: Angiotensin-converting enzyme 2
ARDS: Acute respiratory distress syndrome
ASAT: Aspartate aminotransferase
CCL: C-C motif chemokine ligand
CD: Cluster of differentiation
COVID-19: Coronavirus disease 2019
CXCL: C-X-C motif chemokine ligand
CXCR3: CXC chemokine receptor 3
FLT3L: FMS-like tyrosine kinase 3 ligand
G-CSF: Granulocyte colony-stimulating factor
GM-CSF: Granulocyte-macrophage colony-stimulating factor
ICU: Intensive care medicine
IQR: Interquartile range
IFN: Interferon
IL: Interleukin
IP-10: INF-γ-induced protein 10
MV: Mechanical ventilation
NT-ProBNP: N-Terminal *Fragment* of the Prohormone *Brain*-Type Natriuretic Peptide
PaO_2_: FiO_2_: arterial pressure of oxygen/oxygen inspiratory fraction
PD-L1: Programmed death-ligand 1
RT-PCR: Reverse transcriptase-polymerase chain reaction
SAPS II: Simplified acute physiology score II
SARS-CoV-2: Severe acute respiratory syndrome coronavirus 2
SOFA: Sequential Organ Failure Assessment
TGF: *transforming growth factor*
TNF: Tumor necrosis factor
TRAIL: TNF-related apoptosis inducing ligand
VAP: Ventilator-acquired pneumonia
WBS: Whole blood stimulation
