# Correction to: The dysregulated innate immune response in severe COVID-19 pneumonia that could drive poorer outcome

**DOI:** 10.1186/s12967-021-02746-0

**Published:** 2021-03-08

**Authors:** Mathieu Blot, Jean-Baptiste Bour, Jean Pierre Quenot, Abderrahmane Bourredjem, Maxime Nguyen, Julien Guy, Serge Monier, Marjolaine Georges, Audrey Large, Auguste Dargent, Alexandre Guilhem, Suzanne Mouries-Martin, Jeremy Barben, Belaid Bouhemad, Pierre‑Emmanuel Charles, Pascal Chavanet, Christine Binquet, Lionel Piroth, for the LYMPHONIE Study Group, Pascal Andreu, François Aptel, Marie Labruyère, Sébastien Prin, Guillaume Beltramo, Philippe Bonniaud, Philip Bielefeld, Hervé Devilliers, Bernard Bonnotte, Marielle Buisson, Alain Putot

**Affiliations:** 1 Infectious Diseases Department, Dijon Bourgogne University Hospital, 14 rue Paul Gaffarel, 21079 Dijon, France; 2 Lipness Team, UMR1231 and LabEx LipSTIC, INSERM Research Center LNC, University of Burgundy, Dijon, France; 3 Laboratory of Virology, Dijon Bourgogne University Hospital, Dijon, France; 4 Department of Intensive Care, Dijon Bourgogne University Hospital, Dijon, France; 5 Clinical Epidemiology Unit, INSERM, CIC1432 Dijon, France; 6 Clinical Investigation Center, Clinical Epidemiology/Clinical Trials Unit, Dijon Bourgogne University Hospital, Dijon, France; 7 Anesthesiology and Critical Care Department, Dijon Bourgogne University Hospital, Dijon, France; 8 Dijon Bourgogne University Hospital, Hematobiology, Dijon, France; 9 Cytometry Core Facility, University of Burgundy Franche-Comté, Dijon, France; 10 Department of Pneumology, Dijon Bourgogne University Hospital, Dijon, France; 11 Department of Internal Medicine and Clinical Immunology, Dijon Bourgogne University Hospital, Dijon, France; 12 Department of Internal Medicine and Systemic Diseases, Dijon Bourgogne University Hospital, Dijon, France; 13 Geriatrics Internal Medicine Department, Dijon Bourgogne University Hospital, Dijon, France

## Correction to: J Transl Medi (2020) 18:457 10.1186/s12967-020-02646-9

Following publication of the original article [1], the authors identified an error in the author group. The investigators of the Lymphonie study group weren’t tagged as collaborators. figure are published in this Correction article. The original article has been updated.

The collaborators’ names are included in the ‘Acknowledgements’ section and listed below. The author group has been updated above and the original article [1] has been corrected.

**The investigators of the LYMPHONIE study group are:**

Pascal Andreu, François Aptel, Marie Labruyère, Sébastien Prin, Guillaume Beltramo, Philippe Bonniaud, Philip Bielefeld, Hervé Devilliers, Bernard Bonnotte, Marielle Buisson and Alain Putot.

The publisher apologises to the authors and the readers for the inconvenience caused by this error.
