# Supplementary material for: The dysregulated innate immune response in severe COVID-19 pneumonia that could drive poorer outcome
Source: J Transl Med. 2020 Dec 3;18:457. doi: 10.1186/s12967-020-02646-9 (PMC7711269; doi:10.1186/s12967-020-02646-9)
Supplement: Supplementary file 1 — Additional file 1: Table S1. Microbiological etiologies of severe pneumonia from 63 patients. Table S2. Plasma cytokine concentrations. Table S3. Blood leukocyte cytokine production on ex vivo stimulation. Table S4. Spearman correlation between plasma cytokine concentrations and severity (PaO2:FiO2 ratio, SOFA score) or outcome (duration of mechanical ventilation). Table S5. Spearman correlation between blood leukocyte cytokine production on ex vivo stimulation (Whole Blood Stimulation (WBS)) and severity (PaO2:FiO2 ratio, SOFA score) or outcome (duration of mechanical ventilation). Figure S1. Principal component analysis screen plot (retained dimension = 4) Cattell scree plot criterion retained the first four independent factors; which were clinically interpretable and together preserved 53.47% of all the information contained in the 65 correlated original variables. [file 12967_2020_2646_MOESM1_ESM.docx]

**The dysregulated innate immune response in severe COVID-19 pneumonia that could drive poorer outcome.**

Mathieu Blot, MD, PhD, Jean-Baptiste Bour, PharmD, Jean Pierre Quenot, MD, PhD, Abderrahmane Bourredjem, MSc, Maxime Nguyen, MD, Julien Guy, MD, Serge Monier, PhD, Marjolaine Georges, MD, PhD, Audrey Large, MD, Auguste Dargent, MD, PhD, Alexandre Guilhem, MD, Suzanne Mouries-Martin, MD, Jeremy Barben, MD, Belaid Bouhemad, MD, PhD, Pierre-Emmanuel Charles, MD, PhD, Pascal Chavanet, MD, PhD, Christine Binquet, MD, PhD, Lionel Piroth, MD, PhD, for the LYMPHONIE study group.

-

**Additional Data**

**Additional Table 1. Microbiological etiologies of severe pneumonia from 63 patients**

|  | **Non Covid-19** | | **Covid-19** | | **Total N=63** | |
| --- | --- | --- | --- | --- | --- | --- |
|  | **N=36** | | **N=27** | |  | |
|  | **N** | **%** | **N** | **%** | **N** | **%** |
| **Microbiological pneumonia etiology** |  |  |  |  |  |  |
| Pure Bacterial | 10 | 28% | 0 |  | 10 | 16% |
| *Legionella pneumophila* | 3 | 8% | 0 |  | 3 | 5% |
| *Streptococcus pyogenes* | 2 | 6% | 0 |  | 2 | 3% |
| *Klebsiella pneumoniae* | 1 | 3% | 0 |  | 1 | 2% |
| *Mycoplasma pneumoniae* | 1 | 3% | 0 |  | 1 | 2% |
| *Streptococcus pneumoniae* | 1 | 3% | 0 |  | 1 | 2% |
| *Pseudomonas aeruginosa* | 1 | 3% | 0 |  | 1 | 2% |
| *Staphylococcus aureus* | 1 | 3% | 0 |  | 1 | 2% |
| Pure Viral | 10 | 28% | 26 | 96% | 36 | 57% |
| SARS-CoV-2 | 0 |  | 26 | 96% | 26 | 41% |
| Influenza A | 7 | 19% | 0 |  | 7 | 11% |
| Rhinovirus | 1 | 3% | 0 |  | 1 | 2% |
| Respiratory syncytial virus | 1 | 3% | 0 |  | 1 | 2% |
| Rhinovirus + seasonal coronavirus | 1 | 3% | 0 |  | 1 | 2% |
| Mixed | 3 | 8% | 1 | 4% | 4 | 6% |
| *Haemophilus parainfluenzae* + metapneumovirus | 1 | 3% | 0 |  | 1 | 2% |
| *Staphylococcus aureus* + influenza A | 1 | 3% | 0 |  | 1 | 2% |
| *Streptococcus pneumoniae* + influenza A | 1 | 3% | 0 |  | 1 | 2% |
| *Enterococcus faecium* + SARS-CoV-2 | 0 |  | 1 | 4% | 1 | 2% |
| Other non-documented | 13 | 36% | 0 |  | 13 | 21% |

COVID-19: coronavirus disease 2019; CXCL: C-X-C motif chemokine ligand; CXCR3: CXC chemokine receptor 3; FLT3L: FMS-like tyrosine kinase 3 ligand; G-CSF: Granulocyte colony-stimulating factor; GM-CSF: Granulocyte-macrophage colony-stimulating factor; IFN: interferon; IL: interleukin; IQR: interquartile range; PD-L1: programmed death-ligand 1; TGF*: transforming growth factor*; TNF: tumor necrosis factor; TRAIL: TNF-related apoptosis inducing ligand

**Additional Table 2. Plasma cytokine concentrations (Lymphonie study)**

|  |  | **non-COVID-19** | | **COVID-19** | | **p** |
| --- | --- | --- | --- | --- | --- | --- |
|  |  | **N=36** | | **N=27** | |  |
| **sCD40-Ligand, pg/ml** | **mean ±SE** | 1330.80 | ±1951.86 | 677.50 | ±1002.08 | 0.090 |
|  | **median IQR** | 565.02 | 238.21-1826.16 | 319.47 | 238.21-485.17 | 0.094 |
| **FLT3L, pg/ml** | **mean ±SE** | 210.41 | ±124.69 | 134.97 | ±57.18 | **0.002** |
|  | **median IQR** | 159.71 | 114.17-291.36 | 132.22 | 90.15-171.92 | **0.025** |
| **CX3CL1, pg/ml** | **mean ±SE** | 1683.71 | ±1225.78 | 1344.88 | ±509.79 | 0.141 |
|  | **median IQR** | 1166.35 | 838.81-2058.71 | 1259.24 | 1049.91-1676.11 | 0.912 |
| **G-CSF, pg/ml** | **mean ±SE** | 15044.52 | ±47778.25 | 56.51 | ±56.95 | 0.072 |
|  | **median IQR** | 134.14 | 59.01-1678.88 | 42.84 | 28.73-59.79 | **<.0001** |
| **GM-CSF, pg/ml** | **mean ±SE** | 314.43 | ±236.74 | 429.97 | ±120.72 | **0.015** |
|  | **median IQR** | 288.81 | 72.74-552.89 | 425.92 | 368.69-517.82 | 0.092 |
| **Granzyme B, pg/ml** | **mean ±SE** | 49.87 | ±70.61 | 31.46 | ±20.90 | 0.146 |
|  | **median IQR** | 30.23 | 2.75-69.95 | 31.55 | 18.83-43.62 | 1.000 |
| **CXCL1, pg/ml** | **mean ±SE** | 380.52 | ±846.97 | 93.53 | ±74.81 | 0.051 |
|  | **median IQR** | 121.31 | 80.11-212.76 | 105.21 | 6.69-141.89 | **0.049** |
| **CXCL2, pg/ml** | **mean ±SE** | 647.21 | ±635.29 | 523.51 | ±458.51 | 0.373 |
|  | **median IQR** | 409.53 | 253.03-745.41 | 336.61 | 120.81-745.12 | 0.479 |
| **INF-α, pg/ml** | **mean ±SE** | 11.75 | ±13.52 | 10.28 | ±9.80 | 0.618 |
|  | **median IQR** | 7.20 | 3.90-11.18 | 7.20 | 2.23-17.33 | 0.478 |
| **IL1-α, pg/ml** | **mean ±SE** | 16.50 | ±13.53 | 12.34 | ±9.22 | 0.152 |
|  | **median IQR** | 13.99 | 7.89-17.49 | 10.12 | 4.91-17.49 | 0.094 |
| **IL1-ß, pg/ml** | **mean ±SE** | 4.17 | ±3.73 | 2.49 | ±1.90 | **0.024** |
|  | **median IQR** | 1.77 | 1.77-6.54 | 1.77 | 1.77-1.77 | **0.020** |
| **IL1-RA, pg/ml** | **mean ±SE** | 36100.11 | ±69454.48 | 3987.38 | ±3214.92 | **0.009** |
|  | **median IQR** | 7897.70 | 2451.51-16209.12 | 2881.50 | 1481.80-5826.52 | **0.005** |
| **IL-2, pg/ml** | **mean ±SE** | 13.58 | ±19.84 | 3.38 | ±2.93 | **0.004** |
|  | **median IQR** | 4.50 | 1.80-18.30 | 1.80 | 1.80-4.94 | **0.008** |
| **IL-6, pg/ml** | **mean ±SE** | 7888.39 | ±23397.85 | 199.11 | ±237.98 | 0.057 |
|  | **median IQR** | 460.42 | 138.21-4434.74 | 121.02 | 75.67-236.62 | **0.0003** |
| **IL-7, pg/ml** | **mean ±SE** | 7.52 | ±7.31 | 5.94 | ±3.90 | 0.275 |
|  | **median IQR** | 5.91 | 3.40-7.96 | 5.21 | 2.26-9.61 | 0.601 |
| **IL-8, pg/ml** | **mean ±SE** | 270.55 | ±810.35 | 17.85 | ±10.71 | 0.070 |
|  | **median IQR** | 31.45 | 9.73-106.47 | 13.89 | 11.00-24.55 | **0.030** |
| **IL-10, pg/ml** | **mean ±SE** | 994.37 | ±1441.26 | 1105.60 | ±1577.43 | 0.775 |
|  | **median IQR** | 642.19 | 251.73-1327.71 | 798.16 | 684.31-1020.87 | 0.224 |
| **IL-15, pg/ml** | **mean ±SE** | 13.95 | ±9.53 | 7.49 | ±3.34 | **0.0005** |
|  | **median IQR** | 12.10 | 6.69-20.22 | 7.40 | 4.69-10.14 | **0.0040** |
| **IL-33, pg/ml** | **mean ±SE** | 24.64 | ±16.63 | 25.41 | ±27.61 | 0.898 |
|  | **median IQR** | 21.56 | 12.57-33.53 | 18.03 | 6.87-28.47 | 0.485 |
| **CXCL10, pg/ml** | **mean ±SE** | 4756.69 | ±8515.16 | 4189.28 | ±4722.20 | 0.738 |
|  | **median IQR** | 973.27 | 171.42-4207.74 | 2047.09 | 1354.39-3326.49 | **0.0420** |
| **CCL2, pg/ml** | **mean ±SE** | 1484.81 | ±1717.98 | 704.99 | ±431.42 | **0.012** |
|  | **median IQR** | 624.85 | 378.60-2413.78 | 549.58 | 435.36-859.31 | 0.642 |
| **CCL3, pg/ml** | **mean ±SE** | 40.98 | ±66.09 | 15.37 | ±16.99 | **0.031** |
|  | **median IQR** | 21.43 | 2.49-55.81 | 11.90 | 2.49-21.43 | **0.033** |
| **CCL4, pg/ml** | **mean ±SE** | 2200.34 | ±8451.63 | 397.24 | ±151.38 | 0.203 |
|  | **median IQR** | 488.01 | 340.77-800.99 | 396.94 | 320.63-515.70 | **0.030** |
| **CCL20, pg/ml** | **mean ±SE** | 414.63 | ±511.85 | 91.21 | ±150.13 | **0.0008** |
|  | **median IQR** | 195.07 | 83.26-480.52 | 48.94 | 31.29-67.51 | **<.0001** |
| **CCL19, pg/ml** | **mean ±SE** | 1659.60 | ±5109.46 | 407.76 | ±322.72 | 0.151 |
|  | **median IQR** | 631.69 | 281.78-1337.34 | 335.37 | 212.33-460.06 | **0.014** |
| **PD-L1, pg/ml** | **mean ±SE** | 351.49 | ±207.86 | 315.19 | ±165.85 | 0.444 |
|  | **median IQR** | 299.11 | 197.01-453.66 | 279.14 | 207.52-328.74 | 0.607 |
| **CCL5, pg/ml** | **mean ±SE** | 25039.66 | ±35935.00 | 41976.43 | ±46378.16 | 0.122 |
|  | **median IQR** | 15887.10 | 8507.20-29330.43 | 34842.17 | 12595.23-39278.98 | **0.028** |
| **TGF-α, pg/ml** | **mean ±SE** | 26.52 | ±21.10 | 11.80 | ±9.28 | **0.0005** |
|  | **median IQR** | 18.88 | 14.91-34.17 | 9.19 | 5.79-15.71 | **0.0002** |
| **TNF-α, pg/ml** | **mean ±SE** | 67.84 | ±74.64 | 23.78 | ±24.86 | **0.002** |
|  | **median IQR** | 31.65 | 9.52-107.22 | 15.07 | 8.87-25.26 | **0.019** |
| **TRAIL, pg/ml** | **mean ±SE** | 32.99 | ±46.50 | 18.66 | ±11.80 | 0.083 |
|  | **median IQR** | 15.28 | 10.66-39.96 | 15.28 | 10.66-20.95 | 0.396 |

COVID-19: coronavirus disease 2019; GM-CSF: Granulocyte-macrophage colony-stimulating factor; IFN: interferon; IL: interleukin; IQR: interquartile range; IU: International Unit; TNF: tumor necrosis factor; WBS: whole blood stimulation

**Additional Table 3. Blood leukocyte cytokine production on *ex vivo* stimulation (QuantiFERON Monitor®)**

|  |  | **non-COVID-19** | | **COVID-19** | | **p** |
| --- | --- | --- | --- | --- | --- | --- |
|  |  | **N=36** | | **N=27** | |  |
| **sCD40-Ligand (WBS), pg/ml** | **mean ±SE** | 4298.02 | ±3090.31 | 5158.31 | ±1804.83 | 0.171 |
|  | **median IQR** | 3572.39 | 1714.52-6496.87 | 5151.23 | 3641.56-6425.61 | 0.096 |
| **GM-CSF (WBS), pg/ml** | **mean ±SE** | 69.74 | ±70.55 | 69.84 | ±45.69 | 0.995 |
|  | **median IQR** | 49.67 | 21.64-87.41 | 49.81 | 42.81-91.09 | 0.356 |
| **INF-γ (WBS), IU/ml** | **mean ±SE** | 17.42 | ±29.70 | 17.68 | ±32.86 | 0.974 |
|  | **median IQR** | 2.64 | 0.44-22.12 | 4.42 | 0.70-16.15 | 0.457 |
| **IL1-ß (WBS), pg/ml** | **mean ±SE** | 1339.81 | ±2447.20 | 1108.38 | ±1019.96 | 0.611 |
|  | **median IQR** | 371.59 | 50.46-1862.66 | 768.23 | 424.32-1229.14 | **0.041** |
| **IL-2 (WBS), pg/ml** | **mean ±SE** | 91.03 | ±86.40 | 81.91 | ±41.82 | 0.583 |
|  | **median IQR** | 51.52 | 34.21-120.53 | 68.85 | 54.16-99.61 | 0.314 |
| **IL-4 (WBS), pg/ml** | **mean ±SE** | 8.70 | ±17.07 | 6.89 | ±9.01 | 0.590 |
|  | **median IQR** | 2.64 | 0.60-10.77 | 1.88 | 1.10-7.97 | 0.579 |
| **IL-5 (WBS), pg/ml** | **mean ±SE** | 5.59 | ±11.86 | 4.04 | ±5.23 | 0.489 |
|  | **median IQR** | 1.30 | 0.27-5.60 | 2.16 | 0.00-5.51 | 0.638 |
| **IL-6 (WBS), pg/ml** | **mean ±SE** | 40671.69 | ±44341.75 | 42654.69 | ±27725.90 | 0.829 |
|  | **median IQR** | 28424.75 | 5986.51-63784.38 | 38496.19 | 19777.24-61421.55 | 0.246 |
| **IL-10 (WBS), pg/ml** | **mean ±SE** | 9807.00 | ±11696.41 | 6945.55 | ±6239.75 | 0.217 |
|  | **median IQR** | 7518.07 | 2347.02-12811.24 | 5323.80 | 1807.55-10222.92 | 0.523 |
| **IL-12 (WBS), pg/ml** | **mean ±SE** | 50.83 | ±38.17 | 53.92 | ±19.36 | 0.676 |
|  | **median IQR** | 43.84 | 23.47-70.32 | 52.89 | 38.05-63.20 | 0.204 |
| **IL-13 (WBS), pg/ml** | **mean ±SE** | 56.05 | ±45.63 | 60.89 | ±29.83 | 0.613 |
|  | **median IQR** | 44.95 | 20.19-87.91 | 60.99 | 44.95-78.02 | 0.269 |
| **IL-15 (WBS), pg/ml** | **mean ±SE** | 7.27 | ±4.53 | 8.65 | ±2.52 | 0.127 |
|  | **median IQR** | 7.07 | 3.64-10.58 | 8.38 | 7.28-10.68 | 0.132 |
| **IL-17A (WBS), pg/ml** | **mean ±SE** | 77.41 | ±111.12 | 45.26 | ±64.28 | 0.154 |
|  | **median IQR** | 28.88 | 8.58-90.03 | 20.90 | 10.31-42.39 | 0.697 |
| **IL-33 (WBS), pg/ml** | **mean ±SE** | 46.45 | ±32.00 | 55.87 | ±18.00 | 0.144 |
|  | **median IQR** | 39.89 | 22.59-72.25 | 53.72 | 43.51-70.77 | 0.137 |
| **TNF-α (WBS), pg/ml** | **mean ±SE** | 5362.85 | ±7466.72 | 6178.67 | ±5062.56 | 0.608 |
|  | **median IQR** | 3496.27 | 749.06-7195.63 | 5030.19 | 2648.56-8347.16 | 0.115 |

We used a standardized immune test (i.e. QuantiFERON Monitor®) to measure the cytokine production capacity of whole blood leukocytes on *ex vivo* stimulation (anti-CD3 and TLR7/8 ligand) and thus, immune suppression, in patients with a severe form of CAP (non-COVID-19 and COVID-19), with reference to non-infected control patients (n=7; PNEUMOCHONDRIE study).

COVID-19: coronavirus disease 2019; CXCL: C-X-C motif chemokine ligand; G-CSF: Granulocyte colony-stimulating factor; GM-CSF: Granulocyte-macrophage colony-stimulating factor; IFN: interferon; IL: interleukin; TNF: tumor necrosis factor.

**Additional Table 4. Spearman correlation between plasma cytokine concentration and severity (PaO_2_:FiO_2_ ratio, SOFA score) or outcome (duration of mechanical ventilation).**

|  | **PaO_2_:FiO_2_ ratio** | | **SOFA score** | | **Mechanical ventilation duration** | |
| --- | --- | --- | --- | --- | --- | --- |
|  | **r** | **p** | **r** | **p** | **r** | **p** |
| sCD40-Ligand | -0.11 | 0.38 | 0.19 | 0.13 | 0.20 | 0.11 |
| FLT3L | -0.04 | 0.75 | **0.26** | **0.041** | 0.16 | 0.21 |
| CX3CL1 | 0.02 | 0.89 | **0.25** | **0.045** | **0.29** | **0.023** |
| G-CSF | 0.04 | 0.78 | 0.22 | 0.08 | -0.06 | 0.65 |
| GM-CSF | -0.01 | 0.96 | **0.39** | **0.0017** | **0.49** | **<.0001** |
| Granzyme B | 0.004 | 0.98 | 0.19 | 0.13 | **0.31** | **0.014** |
| CXCL1 | 0.11 | 0.41 | 0.09 | 0.47 | 0.07 | 0.59 |
| CXCL2 | -0.10 | 0.45 | 0.16 | 0.20 | 0.06 | 0.62 |
| INF-α | 0.09 | 0.50 | -0.02 | 0.90 | 0.11 | 0.37 |
| IL1-α | 0.05 | 0.70 | -0.03 | 0.84 | -0.07 | 0.57 |
| IL1-ß | 0.03 | 0.82 | 0.18 | 0.17 | 0.10 | 0.43 |
| IL1-RA | -0.03 | 0.81 | **0.29** | **0.023** | 0.21 | 0.09 |
| IL-2 | -0.04 | 0.78 | **0.26** | **0.038** | -0.095 | 0.46 |
| IL-6 | -0.22 | 0.08 | **0.37** | **0.0027** | 0.21 | 0.11 |
| IL-7 | -0.05 | 0.68 | 0.10 | 0.45 | 0.14 | 0.29 |
| IL-8 | -0.08 | 0.55 | **0.36** | **0.0042** | 0.17 | 0.17 |
| IL-10 | -0.01 | 0.96 | **0.40** | **0.0012** | **0.53** | **<.0001** |
| IL-15 | -0.20 | 0.12 | **0.37** | **0.0029** | 0.22 | 0.084 |
| IL-33 | 0.07 | 0.57 | 0.007 | 0.95 | 0.07 | 0.57 |
| CXCL10 | -0.04 | 0.73 | **0.40** | **0.0013** | **0.54** | **<.0001** |
| CCL2 | -0.18 | 0.16 | **0.42** | **0.0006** | **0.41** | **0.001** |
| CCL3 | 0.14 | 0.29 | 0.08 | 0.52 | -0.06 | 0.64 |
| CCL4 | 0.02 | 0.89 | 0.13 | 0.29 | 0.15 | 0.25 |
| CCL20 | -0.19 | 0.13 | **0.32** | **0.011** | 0.08 | 0.52 |
| CCL19 | -0.11 | 0.37 | **0.39** | **0.002** | 0.13 | 0.31 |
| PD-L1 | -0.01 | 0.96 | **0.34** | **0.007** | 0.19 | 0.13 |
| CCL5 | 0.07 | 0.58 | **-0.28** | **0.02** | -0.01 | 0.94 |
| TGF-α | -0.03 | 0.78 | -0.04 | 0.75 | -0.11 | 0.38 |
| TNF-α | 0.02 | 0.87 | **0.33** | **0.009** | 0.05 | 0.67 |
| TRAIL | 0.08 | 0.54 | -0.11 | 0.41 | -0.006 | 0.96 |

**Additional Table 5. Spearman correlation between blood leukocyte cytokine production on *ex vivo* stimulation (Whole Blood Stimulation (WBS)) and severity (PaO_2_:FiO_2_ ratio, SOFA score) or outcome (mechanical ventilation duration).**

|  | **PaO_2_:FiO_2_ ratio** | | **SOFA score** | | **Mechanical ventilation duration** | |
| --- | --- | --- | --- | --- | --- | --- |
|  | **r** | **p** | **r** | **p** | **r** | **p** |
| sCD40-Ligand (WBS) | 0.03 | 0.81 | **-0.45** | **0.002** | -0.19 | 0.13 |
| GM-CSF (WBS) | 0.02 | 0.85 | **-0.35** | **0.0055** | -0.16 | 0.20 |
| INF-Gamma (WBS) | 0.05 | 0.72 | -0.23 | 0.07 | -0.09 | 0.48 |
| IL1-ß (WBS) | 0.03 | 0.82 | **-0.43** | **0.0004** | -0.15 | 0.23 |
| IL-2 (WBS) | -0.04 | 0.77 | **-0.31** | **0.01** | -0.08 | 0.52 |
| IL-4 (WBS) | -0.03 | 0.84 | **-0.28** | **0.03** | -0.09 | 0.46 |
| IL-5 (WBS) | -0.11 | 0.38 | -0.14 | 0.29 | -0.05 | 0.68 |
| IL-6 (WBS) | 0.00 | 0.99 | **-0.37** | **0.003** | -0.19 | 0.14 |
| IL-10 (WBS) | 0.09 | 0.50 | **-0.50** | **<.0001** | **-0.40** | **0.001** |
| IL-12 (WBS) | 0.12 | 0.35 | **-0.49** | **<.0001** | -0.20 | 0.11 |
| IL-13 (WBS) | -0.08 | 0.51 | **-0.29** | **0.02** | -0.24 | 0.06 |
| IL-15 (WBS) | 0.09 | 0.49 | **-0.45** | **0.0002** | -0.23 | 0.07 |
| IL-17A (WBS) | -0.14 | 0.27 | -0.20 | 0.11 | -0.09 | 0.47 |
| IL-33 (WBS) | 0.03 | 0.83 | **-0.47** | **0.0001** | -0.20 | 0.12 |
| TNF-α (WBS) | 0.02 | 0.90 | **-0.38** | **0.0021** | -0.17 | 0.19 |

COVID-19: coronavirus disease 2019; GM-CSF: Granulocyte-macrophage colony-stimulating factor; IFN: interferon; IL: interleukin; SOFA: Sequential Organ Failure Assessment; TNF: tumor necrosis factor; WBS: whole blood stimulation

**Additional Figure 1. Principal component analysis screen plot (retained dimension = 4)**

**
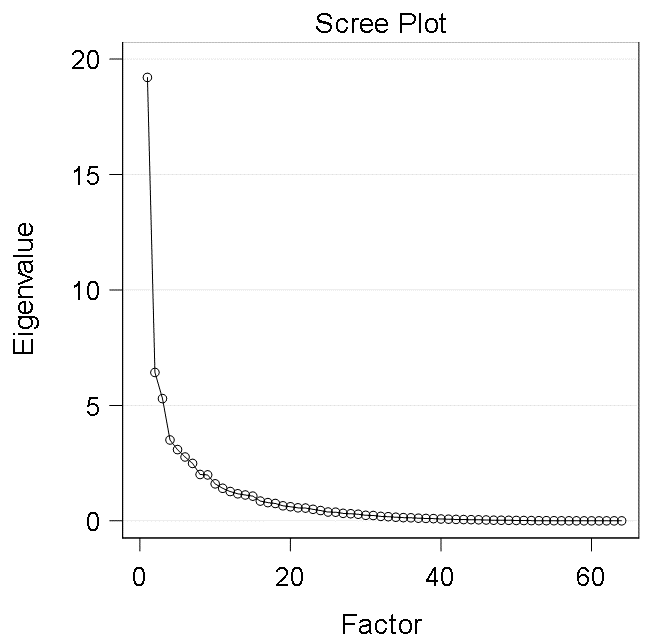
**

Cattell scree plot criterion retained the first four independent factors; which were clinically interpretable and together preserved 53.47% of all the information contained in the 65 correlated original variables.
